# Prognostic Relevance and Function of MSX2 in Colorectal Cancer

**DOI:** 10.1155/2017/3827037

**Published:** 2017-02-12

**Authors:** Jiancheng Liu, Huaying An, Wei Yuan, Qiang Feng, Lianzhen Chen, Jie Ma

**Affiliations:** ^1^State Key Laboratory of Molecular Oncology, National Cancer Center/Cancer Hospital, Chinese Academy of Medical Sciences and Peking Union Medical College, Beijing 100021, China; ^2^Clinical Immunology Center, Chinese Academy of Medical Science, Beijing 100730, China; ^3^Department of Colorectal Surgery, National Cancer Center/Cancer Hospital, Chinese Academy of Medical Sciences and Peking Union Medical College, Beijing 100021, China; ^4^Department of Pharmacy, National Cancer Center/Cancer Hospital, Chinese Academy of Medical Sciences and Peking Union Medical College, Beijing 100021, China; ^5^Beijing Hospital, National Center of Gerontology, Beijing 100730, China

## Abstract

Colorectal cancer patients with diabetes had the high risks of total mortality. High expression of MSX2 is related to development of diabetes. There are few reports about the clinical implications and function of MSX2 in colorectal cancer (CRC). The purpose of this study is to investigate the relationship between the expression of MSX2 and clinical relevance and discover the possible mechanism of MSX2 in the development of CRC. Compared with adjacent tissues, the expression of MSX2 was higher in tumor tissues in both mRNA and protein levels (*P* < 0.01). Kaplan-Meier survival analysis showed that high mRNA expression of MSX2 was associated with short survival time (*P* = 0.013). Chi-squared test analysis indicated that MSX2 expression was related to tumor size (*P* = 0.04), tumor locus (*P* = 0.025), clinical stage (*P* < 0.001), tumor invasion (*P* = 0.003), lymphatic metastasis (*P* = 0.01), and distant metastasis (*P* = 0.033). In vitro experiments demonstrated that knockdown of MSX2 expression attenuated cell proliferation and invasion, promoted cell cycle arrest and apoptosis, and inactivated Akt phosphorylation. In conclusion, MSX2 played a crucial role in the progression of CRC and may be a potential novel prognostic factor and therapeutic target for CRC therapy. Our work may provide certain enlightenment for investigating the mechanism of MSX2 in the process of diabetes.

## 1. Introduction

CRC is the third most commonly diagnosed cancer (1,360,602 new cases) and the fourth cause of cancer mortality (694,000 deaths) worldwide in 2012 [1]. In China, the average number of new cases diagnosed with CRC is 376,300 (the fourth common cancer) each year. The average number of CRC deaths was 191,000 (the fifth common cause of cancer related deaths) in 2009–2011 and the morbidity and mortality of CRC have been increasing [2]. Thus, it is necessary to study the mechanism of CRC to find effective early diagnostic tool and interventions for the therapy of CRC.

Previous studies have indicated that the incidence of colorectal cancer is closely related to genetic factors [3], diet (such as meat, fat) [4], and physical activity [5]. Epidemiological studies have identified a close relationship between diabetes and a higher incidence of CRC. Diabetes increases risk of CRC [6]; CRC patients are more likely to develop diabetes than persons without CRC [7]. Both diabetes and CRC become public health issue around the world.

Muscle segment homeobox genes 2 (MSX2) plays an important role in the development of multiple organs. MSX2-deficient mice showed reduced proliferation of osteoprogenitors, the defects of skull ossification, and persistent calvarial foramen [8]. Studies had shown that high expression of MSX2 was associated with cardiovascular morbidity and mortality in diabetes [9]; cardiovascular disease accounts for 80% of diabetes-related deaths [10, 11].

Increasing evidence indicated that MSX2 was involved in the development of tumor. Clinical studies showed high MSX2 expression was associated with short survival time in prostate [12] and pancreatic cancer patients [13, 14]. MSX2 plays an essential role in gastric cancer growth and knockdown of MSX2 of HSC60 by specific siRNAs significantly inhibited the cell growth [15]. Overexpression of MSX2 promoted the invasion ability of an ovarian epithelial carcinoma cell line (IOSE80) [16]. Study of pancreatic cancer revealed that MSX2 influenced the invasion and migration by inducing the epithelial-mesenchymal transition (EMT) phenotype through upregulation of Twist1 expression [14, 17]. Although current research showed that MSX2 had oncogenic properties and correlated with the high risks of mortality in diabetes, whether it is associated with the development of CRC remains unclear. In this study, we investigated the relationship between MSX2 expression and its clinical relevance, as well as the function of MSX2 in maintaining malignant characteristics of CRC.

## 2. Materials and Methods

### 2.1. Patients and Tissue Specimens

136 pairs of CRC tumor and adjacent tissues were collected from Cancer Hospital, Chinese Academy of Medical Sciences (CAMS), Beijing, China, from March 1, 2011, to November 30, 2011. CRC was confirmed by pathological histology. This research was approved by the medical ethics committee of Cancer Hospital, CAMS, and informed consent was obtained from all patients. After surgery, specimens were freezed immediately in liquid nitrogen within 30 min and then transferred to −80°C refrigerator for later use. Following up was finished in September 30, 2015. Overall survival time was calculated from surgery to death or last observation for surviving patients.

### 2.2. Quantitative Real-Time Polymerase Chain Reaction (qRT-PCR)

The total tissue mRNA was extracted according to standard protocol of the Oligotex mRNA Mini Kit (Qiagen, Hilden, Germany) and then used for reverse transcription by the PrimeScript™ RT Master Mix Kit (Takara, Shiga, Japan). MSX2 and *β*-actin gene primers were designed by primer premier 5.0, and the primers were synthesized by Invitrogen (Invitrogen, California, USA). MSX2 qRT primer sequences were as follows:

MSX2-F (5′-3′): GGAGCGGCGTGGATGCAGGAA

MSX2-R (5′-3′) AAGCACAGGTCTATGGAACGG; *β*-actin-F (5′-3′): AAGGAGCCCCACGAGAAAAAT; *β*-actin-R (5′-3′) ACCGAACTTGCATTGATTCCAG. The annealing temperature of primers is 60°C. Gene expression levels were analyzed using SYBR Premix Ex Taq™ (Takara) on the LightCycler 480 (Roche, Basel, Switzerland). The mRNA level of MSX2 was analyzed by ΔCt method [18], and the relative MSX2 mRNA expression level was normalized to the expression of *β*-actin (ΔCt = Ct MSX2-Ct *β*-actin).

### 2.3. Western Blotting

Total protein was extracted from patient specimens by RIPA buffer (50 mM Tris pH 7.4, 150 mM NaCl, 1% Triton-X100, 1% NP-40, 0.5% sodium deoxycholate, 0.1% SDS) supplemented with a cocktail of protease inhibitors (Sigma). Protein concentration was determined by the method of bicinchoninic acid assay (BCA) (Thermo Scientific). 30 *μ*g total protein was loaded for SDS-PAGE (Bio-Rad, Hercules, CA, USA). After electrophoresis, protein was transferred to nitrocellulose (NC) membrane (GE, Massachusetts, MA, USA), and then the NC membrane was blocked by 5% skim milk at 4°C overnight followed by incubation with the primary anti-MSX2 (1 : 1000 dilution, Abcam, Cambridge, UK) and anti-*β*-actin (1 : 3000, Cell Signaling Technology, CST, Massachusetts, MA, USA) antibody for 1.5 h at room temperature. After that, NC membrane was incubated with horseradish peroxidase-conjugated anti-mouse and anti-rabbit secondary antibodies (1 : 3000, ZSGB-BIO, Beijing, China) for 1.5 h at room temperature. Finally, the protein expression was detected by electrogenerated chemiluminescence (EMD Millipore, Billerica, MA, USA) with ImageQuant-LAS-4000 (GE).

### 2.4. Immunohistochemistry (IHC)

After the surgery, specimens were fixed in 10% formalin, and paraffin-embedded tissues (4 *μ*m thick) were deparaffinized by xylene and rehydrated in the gradient concentrations of alcohol. Heat-induced antigen retrieval was done in the citrate buffer, and endogenous peroxidase was inhibited by 3% H_2_O_2_ at room temperature for 20 min. The slides were then blocked by 5% bovine serum albumin (BSA) (Amresco, Solon, OH, USA) for 1 h at room temperature followed by incubation with mouse monoclonal antibody MSX2 (1 : 200 dilution, Abcam, Cambridge, UK) at 4°C overnight. The sections were then incubated with Goat Anti-Mouse IgG-HRP Secondary Antibody (ZSGB-BIO, Beijing, China) for 30 min followed by diaminobenzidine (DAB) (ZSGB-BIO) color reaction. Antibody dilution buffer omitting primary antibody was used as a negative control. The expression levels of MSX2 in tumor and corresponding normal tissues (*n* = 10) were determined by consideration of both staining intensity and scope.

### 2.5. Cell Culture

The human CRC cell lines HCT-8, HCT-116, LoVo, and SW480 were purchased from American Type Culture Collection (ATCC, Manassas, VA, USA). All the cell lines were maintained in RPMI 1640 Medium (Hyclone, Logan, UT, USA) with 10% fetal bovine serum (Sigma, St. Louis, MO, USA), 100 U/mL penicillin, and 100 *μ*g/mL streptomycin at 37°C in 5% CO_2_ incubator (Thermo Scientific, Massachusetts, MA, USA).

### 2.6. Small Interfering RNA Transfection

For knockdown of MSX2 expression, specific MSX2 small interfering RNA (siRNA), and negative control (NC) sequences were designed and synthesized by Ribobio Co. Ltd. (Ribobio, Guangzhou, China). The oligonucleotide sequences were shown as follows: MSX2 siRNA sense sequence (5′-UGAGGAAACACAAGACCAAdTdT-3′); antisense sequence (5′-UGAGGAAACACAAGACCAAdTdT-3′). An unrelated scrambled siRNA was served as a negative control. The cell lines were transfected with a final concentration of 50 nM siRNA according to manual of the siRNA transfection kit (Ribobio), and function assays were performed after 48 h transfection.

### 2.7. Cell Proliferation Assays

Cancer cells were transfected with small interfering negative control RNA (si-NC) or specific MSX2 small interfering RNA (si-MSX2), respectively, and harvested after 48 h transfection. Cell proliferation assays were detected by MTT. Transfected cells (1 × 10^3^ cells in 100 *μ*L medium per well) were seeded in 96-well plate (Corning, New York, NY, USA) with five repetitive wells. 50 *μ*L MTT (500 *μ*g/mL) was added in the detected wells every day and then cells were cultured in 37°C incubator for 2 h. After incubation, the medium with MTT was discarded. 100 *μ*L DMSO was added in each well followed by slowly shaking on the vortex for 10 min. The absorbance was measured in a microplate reader at 490 nm wavelength (BioTek, Winooski, VT, USA).

### 2.8. Cell Cycle and Apoptosis Assays

For apoptosis detection, transfected cells were staining with 7AAD and Annexin V-PE (Bio-Box, Nanjing, China). For cell cycle detection, transfected cells were fixed in 70% ethanol overnight at 4°C and then washed with phosphate buffered saline (PBS). After washing, cells were resuspended in 500 *μ*L PBS with PI (50 mg/L) and RNaseA (100 mg/L) (Sigma, St. Louis, MO, USA) and analyzed with a Becton Dickinson (BD) flow cytometry instrument (BD, New Jersey, USA). FlowJo software (FlowJo, Ashland, OR, USA) was used for cell cycle and apoptosis analysis.

### 2.9. Cell Migration and Invasion Assays

Invasion and migration assays were performed using 24-well chambers (pore size 8 *μ*m) either uncoated (for migration) or coated with 50 *μ*L matrigel (0.2 *μ*g/*μ*L) (for invasion). The cells were starved for 24 h with serum-free medium and then seeded in the upper chamber in 300 *μ*L serum-free RPMI medium (1 × 10^5^ cells). Complete RPMI medium with 10% FBS (600 *μ*L) (Sigma) was added to the lower chamber as chemoattractant. After incubation for 24 h at 37°C, 5% CO_2_, cells were washed by wet cotton-tipped and fixed in methanol for 20 min followed by staining with 1% crystal violet for 20 min. The cells penetrated were photographed (×200) and counted.

### 2.10. Statistical Analysis

Statistical analyses were performed by IBM SPSS Statistics 19.0 (SPSS, Chicago, IL, USA). Pearson's Chi-square test was used to analyze the correlation between MSX2 expression and clinicopathological parameters. The relationship between the overall survival rate and the MSX2 expression difference was evaluated by Kaplan-Meier method with log-rank test. The difference between groups was determined by two-tailed *t*-test and the statistical significance was defined when* P* value is less than 0.05. *∗* denotes *P* values < 0.05, *∗∗* denote *P* value < 0.01, and ∗∗∗ denote *P* value < 0.001.

## 3. Results

### 3.1. MSX2 Was Highly Expressed in CRC Tissues

**Figure 1:**
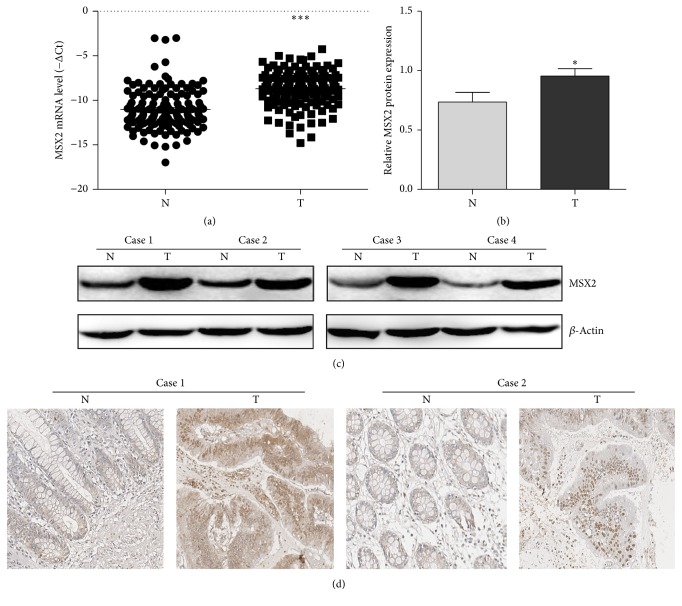
The expression of MSX2 in CRC tumor tissue (T) and adjacent tissue (N). (a) The MSX2 expression of T and N in 136 CRC patients was determined by qRT-PCR (^*∗∗∗*^*P* < 0.001, *n* = 136). Lower ΔCt value indicates higher MSX2 expression (ΔCt = CtMSX2 − Ct*β*-actin). (b) The protein expression of MSX2 of T and N was determined by western blotting (^*∗*^*P* < 0.05, *n* = 10). The MSX2 expression was normalized to *β*-actin. (c) Representative western blotting analysis of MSX2 expression in T and N and *β*-actin was used as a loading control. (d) Representative immunohistochemical analysis of MSX2 expression in T and N.

To identify potential CRC related genes, we evaluated MSX2 expression in primary CRC tissues and adjacent tissues by qRT-PCR, IHC, and western blotting. In 136 CRC patients, the expression of MSX2 mRNA in tumor tissues had a higher level than that in adjacent tissues (ΔCt value −8.6921 ± 1.97 versus −11.02 ± 2.29, *P* < 0.001) Figure 1(a). Consistently, western blotting confirmed the upregulation of MSX2 in tumor tissues at protein level (*P* < 0.01, *n* = 10; Figures 1(b) and 1(c)). Among these results, four representative cases were showed in Figure 1(b). An IHC analysis with another 10 pairs of sample was further performed to validate the expression of MSX2 in colorectal tissue, which showed a stronger expression in tumor tissues compared with the corresponding adjacent tissues in 7 pairs. Two representative cases were showed in Figure 1(d). Collectively, these results indicated that MSX2 expression was significantly elevated in CRC tumor tissues compared with adjacent tissues.

### 3.2. The Relationship between MSX2 Expression and Clinical Relevance

**Figure 2:**
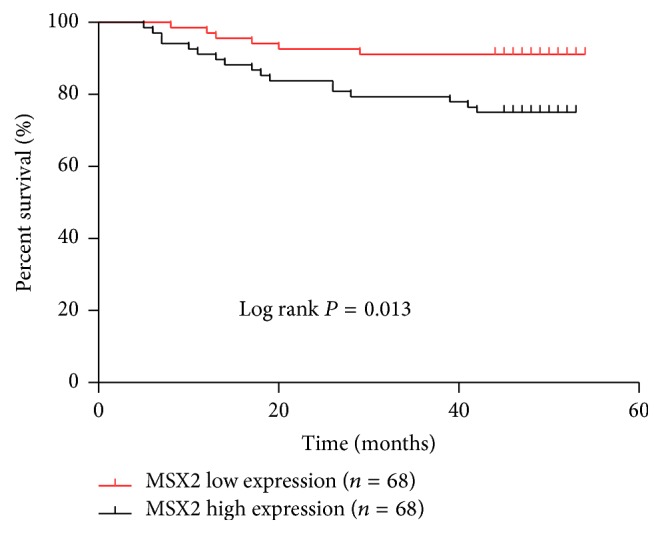
Kaplan-Meier survival analysis and log-rank test for overall survival of 136 CRC patients according to MSX2 mRNA expression level. Higher MSX2 expression predicated a shorter survival time (log rank, *P* = 0.013). Red and black lines represent lower and higher MSX2 expression, respectively.

To determine the clinical significance of MSX2 in CRC, the relationship between the mRNA expression of MSX2 and clinical characteristics was investigated by Chi-squared test. Since the median ΔCt of MSX2 in tumor tissues was 8.50, the 136 patients were divided into high and low expression groups according to ΔCt value less than 8.5 or greater than 8.5, respectively. The Chi-squared test showed that high MSX2 mRNA expression was associated with tumor locus (*P* = 0.025), tumor size (*P* = 0.040), clinical stage (*P* < 0.001), tumor invasion (*P* < 0.001), lymphatic metastasis (*P* = 0.01), and distant metastasis (*P* = 0.033). By contrast, there was no association between MSX2 mRNA expression and gender (*P* = 0.600), age (*P* = 0.123), or tumor differentiation (*P* = 0.683) (Table 1). Kaplan-Meier survival analysis was carried out to investigate the association between MSX2 expression and the survival. The total 54-month survival rate of low MSX2 expression group was 89.7%, while the high MSX2 expression group was 76.4%. The statistical results showed that high MSX2 expression was significantly associated with short survival time (log-rank test, *P* = 0.013, *n* = 136) (Figure 2). Overall, these results indicated that high expression of MSX2 was correlated with aggressive clinical features and poor prognosis.

### 3.3. Knockdown of MSX2 Expression Inhibited the Cell Proliferation

**Figure 3:**
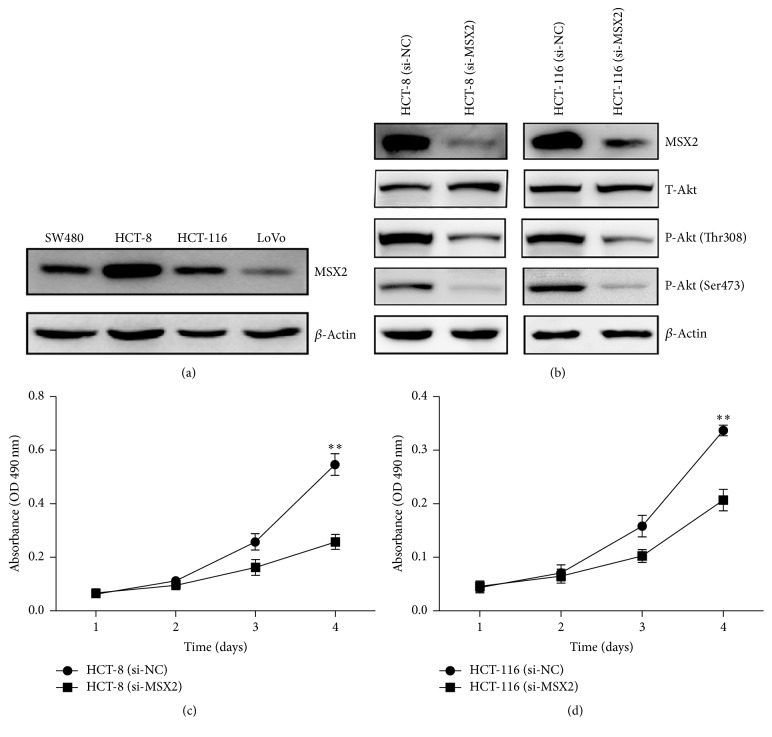
Knockdown of MSX2 expression suppressed proliferation of the CRC cell lines. (a) The expression of MSX2 was analyzed by western blotting in different CRC cell lines (SW480, HCT-8, HCT-116, and LoVo) and *β*-actin was used as a loading control. (b) Expression of MSX2, T-Akt, and P-Akt in HCT-116 and HCT-8 transfected with si-NC or si-MSX2 was detected by western blotting and *β*-actin was used as a loading control. MTT assay was used to analyze the proliferation of CRC cells transfected with si-NC or si-MSX2 in HCT-116 (c) and HCT-8 cells (d) (^*∗∗*^*P* < 0.01). Data show the mean ± SD of five independent experiments at each time point.

The above clinical analysis indicated that high MSX2 expression was associated with the tumor size and clinical stage, which prompted us to explore the function of MSX2 in CRC cells. We examined the expression of MSX2 in different CRC cell lines (HCT-8, HCT-116, SW480, and LoVo) by western blotting. In HCT-8 and HCT-116 cell lines, we observed high expression of MSX2 (Figure 3(a)). Thus, we chose these two cell lines to study the biological role of MSX2. The expression of MSX2 was significantly decreased in HCT-8 and HCT-116 cells after the transfection with specific MSX2 small interfering RNA (si-MSX2) (Figure 3(b)). The knockdown of MSX2 expression resulted in decreased proliferation of HCT-8 and HCT-116 cells (Figures 3(c) and 3(d)). We further investigated the potential mechanism by which MSX2 affected cell proliferation. Akt signal pathway, which plays a crucial role in regulating cell proliferation and differentiation [19, 20], was analyzed by western blotting. Although there was no difference of total Akt expression in HCT-8, HCT-116 cell lines transfected with si-MSX2 compared to nontargeting control (si-NC), a significant reduction of Akt activation was observed (Figure 3(b)). Collectively, these results indicated that MSX2 promoted CRC cell proliferation through activation of Akt signal pathway.

### 3.4. Knockdown of MSX2 Expression Promoted Cell Cycle Arrest and Cell Apoptosis

**Figure 4:**
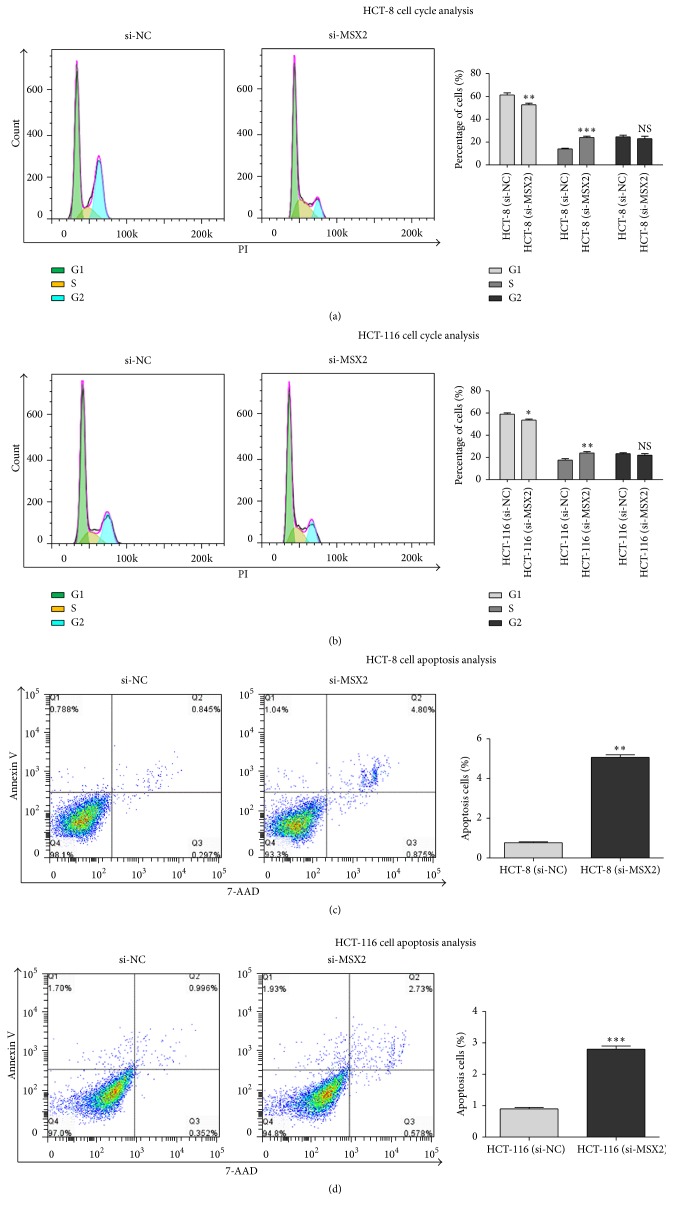
Knockdown of MSX2 expression promoted the cell cycle arrest and apoptosis of CRC cell line. HCT-8 (a, c) and HCT-116 cells (b, d) were harvested after transfection with si-MSX2 and si-NC for 48 h. (a, b) Representative histograms of cell cycle distribution detected by flow cytometry for PI-staining. The green, blue, and yellow peaks represent G1 stage, G2 stage, and S stage, respectively (left panel); data summary of the proportion of cell cycle distribution (right panel) (^*∗*^*P* < 0.05, ^*∗∗*^*P* < 0.01, ^*∗∗∗*^*P* < 0.001, compared with NC). (c, d) Representative dot-plots of apoptosis detection by flow cytometry for Annexin V-PE and 7-AAD staining (left panel); data summary of the proportion of apoptotic cells with Annexin V^+^7-AAD^+^ cells (right panel) (^*∗∗*^*P* < 0.01, ^*∗∗∗*^*P* < 0.001, compared with NC). Error bar value represented at least 3 independent experiments (mean ± SD).

Since cell proliferation was often related to the cell cycle and apoptosis, we analysed the effect of MSX2 on cell cycle distribution and the ratio of apoptotic cells by flow cytometry. Knockdown of MSX2 expression resulted in S phase cell arrest, an increase of the cell proportion in G1 phase in HCT-116 and HCT-8 (Figures 4(a) and 4(b)). Apoptotic cell was detected by flow cytometry with Annexin v/7AAD double labeling. The ratio of the apoptotic cells (Annexin v/7AAD double positive) in HCT-116 and HCT-8 transfected with si-MSX2 was lower than that with si-NC (Figures 4(c) and 4(d)). These results suggested that knockdown of MSX2 expression induced the CRC cell cycle arrest and apoptosis.

### 3.5. Knockdown of MSX2 Expression Inhibited the Migration and Invasion of CRC Cell

**Figure 5:**
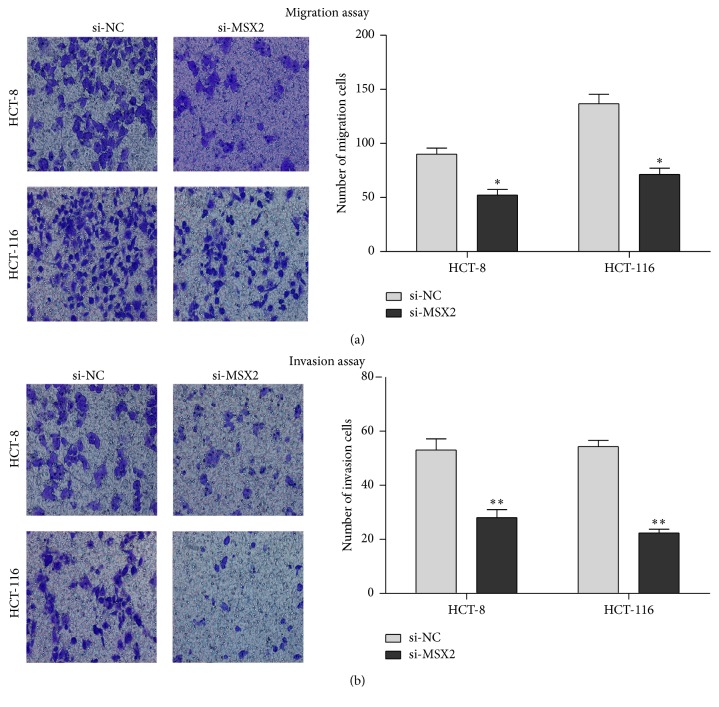
Knockdown of MSX2 expression suppressed the migration and invasion of CRC cell line in vitro. Representative photomicrographs of migration (chamber uncoated with matrigel) (a) and invasion (chamber coated with matrigel) (b) of HCT-8 and HCT-16 cell lines (left panel) and data summary of the number of cells that penetrated the transwell (right panel) (^*∗*^*P* < 0.05, ^*∗∗*^*P* < 0.01 compared with NC). Original magnification, ×200. Data show mean ± SD of at least 3 independent experiments.

The analysis of clinical samples indicated that high MSX2 expression was related to lymphatic metastasis and distant metastasis. Therefore, we performed a transwell assay to verify the function of MSX2 in regulating cell migration (chamber with no matrigel coating) and invasion (chamber with matrigel coating). The counting of the cells penetrated transwell chamber indicated that knockdown of MSX2 expression significantly decreased the abilities of migration and invasion of HCT116 and HCT-8 cell lines (Figure 5). Overall, these transwell assays indicated that knockdown of MSX2 expression reduced migration and invasion of CRC cell in vitro.

## 4. Discussion

In order to verify whether MSX2 was correlated with CRC, we evaluated the expression of MSX2 in 136 CRC patients. We observed a higher expression of MSX2 in CRC tumor tissues than adjacent tissues in mRNA and protein level, which correlated with the malignancy of CRC. High expression of MSX2 was associated with the poor clinical prognosis. Our results were consistent with previous reports about prostate and pancreatic cancer. In prostate cancer, the expression of MSX2 was upregulated in the tumors with bone metastasis. The higher expression of MSX2, the poorer metastasis-free survival in patients. MSX2 was considered to be an independent prognostic factor in advanced prostate carcinoma [12].

Among the 136 pairs of CRC samples, 113 had significant higher expression in tumor tissues compared with corresponding adjacent tissues. Since MSX2 generally plays a regulatory role in the protein level, we evaluated the protein expression of tumor tissues with increased mRNA expression using western blotting. The result showed a consistent expression in mRNA and protein level. Another compliment experiment with 10 pairs of samples by IHC showed that 7 of which had significant high expression in tumor tissues, which further confirmed high expression of MSX2 protein in CRC. Although these results are needed to be validated in large scale CRC samples, our work provided certain enlightenment for analyzing the relationship between protein level of MSX2 expression and the clinical relevance in the future.

In order to clarify the mechanism behind the phenomenon that high MSX2 expression was correlated with poor prognosis in the CRC, we knocked down the expression of MSX2. The deficiency of MSX2 resulted in inhibition of cell proliferation, cell cycle arrest, apoptosis, and reduced migration and invasion. Since Akt signaling pathway has been reported as an important player in the development of CRC [21]. We investigated the change of Akt in MSX2 knockdown CRC cell lines. Although there was no change in total Akt expression, a correlation between MSX2 and Akt phosphorylation was evidenced in HCT-8 and HCT-116 cell lines by western blotting. Previous studies revealed that the phosphorylation of Akt promoted CRC cell proliferation and cycle progression through inactivation of p21 [22]. Akt activation also inhibited cell apoptosis independent on p53 pathway [23]. Additionally, Akt signaling increased the invasion ability via upregulation of MMP7 in CRC [24]. These evidences indicated that MSX2 could regulate the malignant development of CRC through Akt pathway. Recent studies suggested that advanced glycation end products (AGEs) induced vascular calcification by activating the Akt signaling pathway [25]. Whether MSX2 induce vascular calcification in diabetic patients by Akt signaling pathway is worth further study.

WNT/*β*-catenin is another signaling pathway associated with MSX2 expression. In the ovarian cancer cell lines, MSX2 expression was induced by WNT/*β*-catenin signaling, which had been considered as the contributor to the malignancy of ovarian cancer. In the diabetic animal model, MSX2 promoted vascular calcification by upregulation of Wnt 3a and Wnt 7a expression [9]. Although aberrant activation of Wnt signal is one of the important causes of CRC [26], we hypothesize whether there is a positive feedback regulation loop between MSX2 and Wnt signaling pathways during the process of CRC and diabetes. More studies are needed to disclose MSX2 centered network in life activities.

The characteristics of MSX2 affecting the migration and invasion of pancreatic cancer cell were also reflected in the appearance of EMT phenotype [14]. In CRC, the changes of cell morphology and EMT marker did not accompany MSX2 expression in HCT-8 and HCT-116 (data not shown). Thus, the exact mechanism by which MSX2 promotes CRC progression needs further study.

Tumor microenvironment has abundant blood vessels which is an important channel for tumor nutrition and escape. Therefore, our hypothesis is that MSX2 may promote the distal metastasis of the tumor cells via vascular calcification. Previous report indicated that inflammation promoted tumor metastasis [27]. Calcified vascular area has inflammation and damage of vascular endothelial and may provide a good chance of escape for tumor cell [28], which needs further investigation.

## 5. Conclusion

In summary, this is the first investigation of the correlation of MSX2 and CRC. The study demonstrated that high expression of MSX2 is associated with short survival time in CRC patients. MSX2 promoted proliferation and invasion of CRC cells through the Akt signaling pathway. Our work indicated that MSX2 may be an important prognostic marker and a potential therapeutic target in CRC, which may expand our understanding of the potential mechanism of MSX2, affecting the vascular calcification in diabetes.

## Figures and Tables

**Table 1:** Relationship between the MSX2 expression and clinicopathological characteristics of CRC.

Characteristics	Num	MSX2 expression		*X* ^2^	*P* value^a^
		Low	High		
Gender				0.275	0.600
Male	81	39	42		
Female	55	29	26		
Age (years)^b^				2.383	0.123
<60	67	38	29		
≥60	69	30	39		
Tumor locus				5.024	**0.025**
Rectum	75	44	31		
Colon	61	24	37		
Tumor size^b^				4.239	**0.040**
<4.5 cm	70	39	27		
≥4.5 cm	66	29	41		
Tumour differentiation				0.762	0.683
Well	17	10	7		
Moderate	101	50	51		
Poor	18	8	10		
Clinical stage				24.846	<**0.001**
I	27	20	7		
II	29	20	9		
III	65	25	40		
IV	15	3	12		
Tumor invasion				8.815	**0.003**
T1 + T2	28	8	0		
T3 + T4	108	13	7		
Lymphatic metastasis				14.576	**0.010**
L0	58	40	18		
L1	40	14	26		
L2	38	14	24		
Distant metastasis				4.533	**0.033**
Yes	16	4	12		
No	120	64	56		

*Notes*. ^a^Pearson's Chi-square test; *P* < 0.05 was defined for statistical significance. ^b^Median value.
